# A modular, mechanism-informed biomarker panel for asthenozoospermia based on integrative sperm proteomics

**DOI:** 10.3389/fendo.2026.1850544

**Published:** 2026-08-10

**Authors:** D. Lorenzi, L. Cuniolo, D. J. Cohen, V. G. Da Ros

**Affiliations:** 1 Laboratorio de Biología de las Gametas, Instituto de Biología y Medicina Experimental (IBYME), Fundación IBYME, Consejo Nacional de Investigaciones Científicas y Técnicas (CONICET), Ciudad Autónoma de Buenos Aires, Argentina; 2 Universidad Argentina de la Empresa (UADE), Instituto de Tecnología (INTEC), Ciudad Autónoma de Buenos Aires, Argentina

**Keywords:** asthenozoospermia, biomarker panel, male infertility, proteomics, sperm

## Abstract

**Introduction:**

Asthenozoospermia (AZS), defined by reduced sperm motility, is a major cause of male infertility. Comparative sperm proteomic studies of AZS versus normozoospermic men have reported differential protein expression; however, their biological interpretation remains fragmented and largely centered on individual proteins.

**Methods:**

We performed an integrative analysis of 16 independent human sperm proteomic datasets using a two-step recurrence-based strategy to identify robust AZS-associated molecular signatures. Differentially expressed proteins (DEPs) reported in at least two independent studies were considered recurrent and subjected to Gene Ontology, KEGG, Reactome, and Human Phenotype Ontology (HPO) enrichment analyses. The most highly recurrent DEPs within each direction of regulation (upregulated and downregulated) were further selected as core DEPs and analyzed through protein–protein interaction network analysis.

**Results:**

Among 2,459 non-redundant DEPs, 701 proteins (259 upregulated and 442 downregulated in AZS) were recurrently identified across studies, representing approximately 29% of all reported proteins. Recurrently upregulated proteins were primarily associated with proteostasis-related processes, RNA-related pathways, vesicle-mediated trafficking, and extracellular seminal plasma components, whereas recurrently downregulated proteins were linked to mitochondrial energy metabolism and flagellar structure. Protein–protein interaction network analysis of the core DEPs, integrating both upregulated and downregulated sets, revealed four conserved functional modules across sperm compartments: structural, metabolic, vesicular trafficking, and extracellular.

**Discussion:**

These findings show that molecular alterations in AZS converge at the level of functional modules rather than representing isolated protein-level dysregulation. This modular architecture defines a mechanism-informed biomarker panel that captures key processes underlying AZS, helps reconcile discrepancies among studies, and provides a framework for molecularly grounded diagnostics and future validation through multi-omics and artificial intelligence approaches.

## Introduction

Approximately 10% of couples worldwide experience infertility, with male factors accounting for about 50% of cases (1). Male infertility is currently diagnosed through physical examination, clinical history, hormonal profiling, and semen analysis. Reduced sperm motility, termed asthenozoospermia (AZS) (defined as progressive sperm motility <32% and/or total motility <40%; 2), affects around 40–50% of infertile men. Its molecular basis remains poorly understood, partly due to its multifactorial nature. Consequently, clinical management largely relies on intracytoplasmic sperm injection (ICSI), a technical solution that bypasses, rather than addresses, the underlying molecular defects. Although effective for achieving fertilization and early embryo development, improvements in live birth rates remain modest (3). Moreover, ICSI may increase the risk of transmitting genetic and/or epigenetic abnormalities, as it circumvents physiological selection processes (4). These limitations highlight the need for a deeper molecular understanding of AZS to enable a transition from phenotype-based diagnosis to mechanism-informed strategies.

This clinical phenotype is fundamentally rooted in the complex biological machinery for sperm movement. Sperm motility depends on both the structural and functional integrity of the flagellum — a highly conserved structure composed of an axonemal core, associated accessory components, mitochondria, and motor and regulatory proteins (5, 6) — and coordinated biochemical maturation events. During epididymal transit, sperm acquire motility competence through extensive remodeling driven by the luminal environment, involving intracellular signaling, metabolic activation, and post-translational modification of flagellar proteins (7). After ejaculation, sperm undergo capacitation within the female reproductive tract, acquiring the hyperactivated motility required for fertilization. Because mature sperm exhibit limited capacity for *de novo* protein synthesis (8, 9), their function mainly depends on a pre-established proteome (10), making protein composition a key determinant of motility and an indicator of functional competence. This unique biological feature renders sperm proteomic analysis particularly well-suited to investigate the molecular basis of AZS.

In the last decades, different research groups have performed independent comparative proteomic analyses between AZS and normozoospermic (NZS) sperm samples, generating a substantial body of isolated protein lists. As a consequence, several studies have compiled these datasets to identify candidate protein panels associated with sperm motility (11–13). However, the rapidly evolving proteomic field promotes the continuous revision of existing data. Moreover, the biologically integrated interpretation of AZS beyond the conventional upregulated/downregulated protein classification is required to generate new insights into its etiology (14).

To address this, we performed an updated integrative analysis of published human sperm proteomic datasets. By applying a recurrence-based strategy combined with protein–protein interaction network analysis, we show that molecular alterations associated with AZS are not randomly distributed but consistently organized into four major functional modules—structural, metabolic, vesicular trafficking, and extracellular. This modular architecture, rather than any individual protein signature, offers a mechanism-informed framework to complement traditional semen analysis and potentially improve patient stratification in assisted reproduction.

## Methods

### Selection of human sperm proteomic datasets

A structured literature search was conducted in July 2025 using the PubMed database of the National Center for Biotechnology Information (NCBI) (https://pubmed.ncbi.nlm.nih.gov/) and the PRIDE database of the European Bioinformatics Institute (EMBL-EBI) (https://www.ebi.ac.uk/pride/), which contains mass spectrometry-based proteomics datasets. Searches included the keywords “asthenozoospermia proteomic” or “male infertility sperm proteomic” and were restricted to humans and English language. Publications that reported comparative sperm proteomic datasets between AZS and NZS individuals were considered eligible. Sample preparation method and proteomic approach for each study were documented. Reports were excluded if they: i) used swim-up-selected AZS sperm samples prior to proteomic analysis; ii) were based exclusively on seminal plasma or exosomes analysis; iii) involved patients with additional clinical conditions (e.g., obesity or cancer); or iv) only compiled previous proteomic articles. All included studies defined AZS according to World Health Organization (WHO) criteria, based on either the 1999 (progressive motility < 25% or total motility < 50%) (15) or the 2010 (progressive motility < 32% or total motility < 40%) (2) editions.

### Data extraction and curation

Protein data were retrieved from the main text and Supplementary Materials of the selected publications. Differentially expressed proteins (DEPs) between AZS and NZS individuals were identified based on the statistical criteria reported in each study; no re-analysis of raw data was performed. Only proteins reported as statistically significant with *p*-value < 0.05 were included, with some studies applying additional filters (e.g., FDR-based criteria or fold-change thresholds ranging from 1.2 to 2). Proteins were classified as upregulated (UR) or downregulated (DR) in AZS patients as originally described. To enable cross-study comparison, protein identifiers were manually curated and harmonized using UniProt accession codes, protein names, and corresponding gene symbols. Entries lacking gene annotation or not corresponding to human proteins were excluded. Synonymous gene names mapping to the same UniProt accession were unified, and outdated or uncharacterized protein annotations were updated when possible. Following harmonization, non-redundant datasets were generated by retaining a single entry per protein and recording the number of independent studies in which each protein was reported. The curated protein list was processed and plotted in R (version 4.5.2) using the *dplyr* package.

### Functional enrichment and pathway analysis

Only DEPs consistently reported as either UR or DR in at least 2 studies were included, and each list was analyzed separately. HGNC (Hugo Gene Nomenclature Committee) symbols were used as input with default parameters.

Gene Ontology (GO) enrichment analysis was performed using the ToppFun tool of the ToppGene Suite (https://toppgene.cchmc.org/) (16). Enriched molecular functions, biological processes, and cellular components were identified, and statistical significance was determined using the hypergeometric probability mass function with Benjamini–Hochberg false discovery rate (FDR) correction (adjusted *p*-value < 0.05). GeneRatio was defined as the proportion of input proteins associated with each GO term relative to the total number of proteins analyzed in the corresponding list.

Pathway Overrepresentation Analysis (ORA) was conducted using the Reactome Pathway Browser (https://reactome.org, version 96) (17). Significantly enriched pathways were identified using a hypergeometric test with Benjamini–Hochberg FDR correction (adjusted *p*-value < 0.05).

Complementary enrichment analysis was carried out using g:Profiler (https://biit.cs.ut.ee/gprofiler/gost, version e112_eg59_p18) (18) with g:SCS multiple testing correction method (adjusted *p*-value < 0.05) (19), querying the Kyoto Encyclopedia of Genes and Genomes (KEGG) pathway database (20) and the Human Phenotype Ontology (HPO) (21).

In all cases, data visualization was performed in RStudio using the *ggplot2* package.

### Protein–protein interaction analysis

Protein–protein interaction (PPI) networks for UR and DR proteins belonging to the highest recurrence categories within each list were generated using STRING (version 12, https://string-db.org/) (22). An additional network was constructed using a pooled set of the highly recurrent proteins regardless of their UR or DR classification. All analyses were performed for *Homo sapiens* with a medium confidence threshold (interaction score > 0.4). The full STRING network was used, incorporating both physical and functional associations derived from experimental data, curated databases, co-expression, gene neighborhood, gene fusion, co-occurrence, and text mining. Edges represent the evidence supporting each interaction, with edge thickness reflecting the strength of the supporting data.

### Characterization of candidate proteins

Targeted literature searches were conducted in PubMed to identify studies describing protein abundance and function in human sperm or, when unavailable, in mammalian models.

## Results

**Figure 1:**
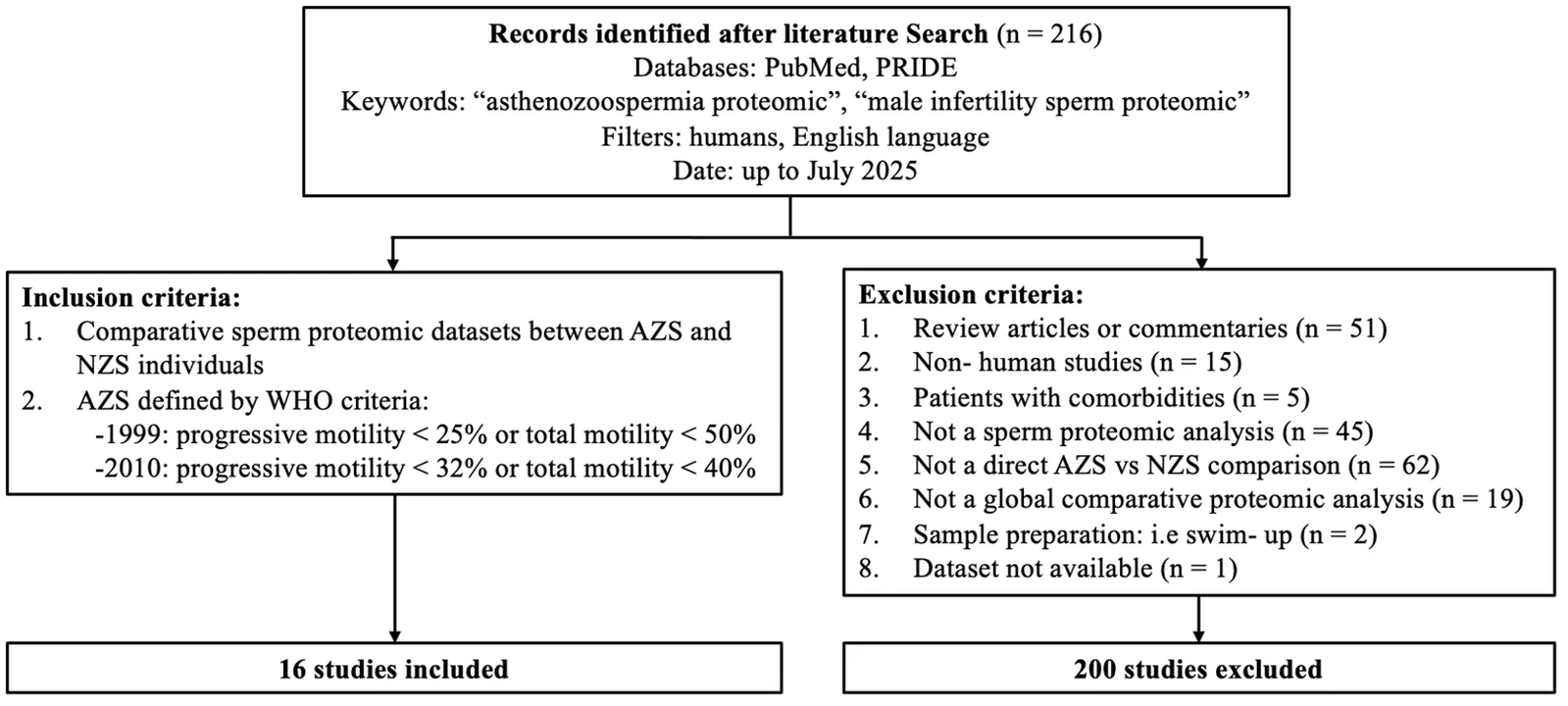
Flowchart diagram showing the article selection process for the integrative analysis.

**Figure 2:**
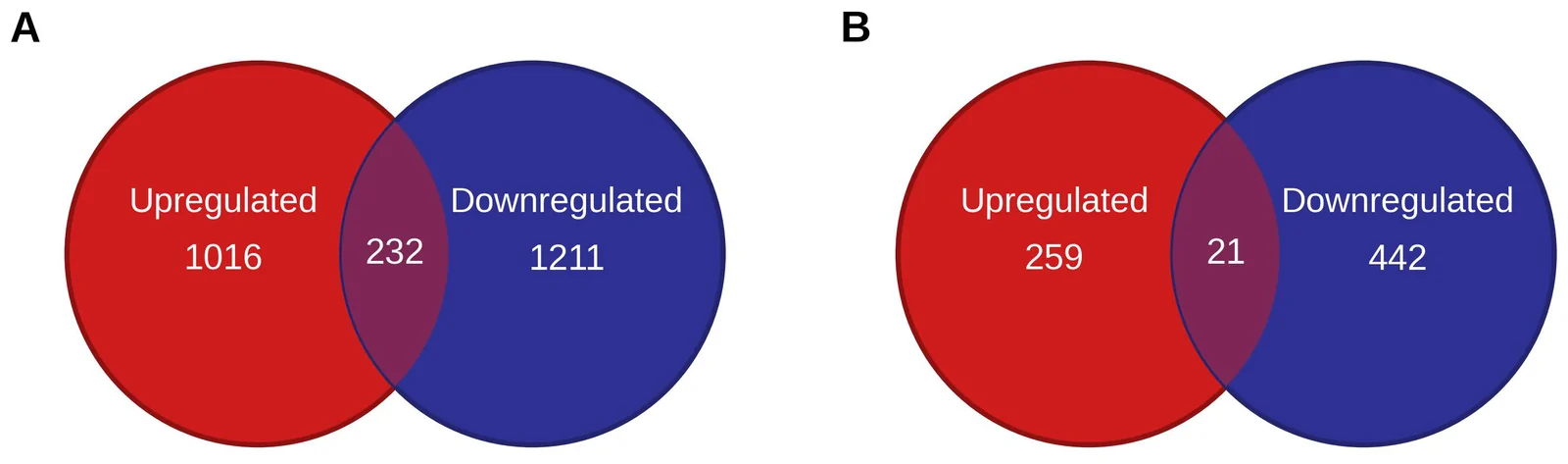
Differentially expressed sperm proteins in AZS relative to NZS. **(A)** Venn diagram summarizing all proteins reported as differentially expressed across the studies included in the analysis. The overlapping region indicates proteins reported as upregulated (UR) in some studies and downregulated (DR) in others, irrespective of the number of supporting studies. **(B)** Venn diagram showing proteins recurrently identified as differentially expressed in ≥ 2 independent studies. The overlapping region represents proteins reported as UR and DR in different studies, with ≥ 2 studies supporting one direction and ≥ 2 the opposite.

A schematic representation of the study selection process is shown in **Figure 1**. Among the studies identified through the literature search, 16 reports comparing AZS vs NZS sperm proteomics were included in this integrative analysis (**Table 1**) whereas 200 were excluded (**Supplementary Table 1**). After compilation, curation and harmonization of protein identifiers, 3,702 DEPs were identified (**Table 1**; **Supplementary Figure 1**). A non-redundant list of 2,459 unique DEPs from AZS vs NZS comparisons was generated by retaining a single entry for each protein. Of these, 1,016 were originally classified as UR and 1,211 as DR, while 232 were inconsistently reported as both UR and DR in different studies (**Figure 2A**).

**Table 1:** Studies included in the integrative proteomic analysis.

Study	Clinical condition of the patients	Selection method	Study groups (N)		Mass spectrometry method	N° of DEPs	
			NZS	AZS		UR	DR
Zhao et al., 2007 (23)	Rapid motility: 0-3% and progressive motility: 5-20% *	1-layer Percoll gradient	8	8	2D electrophoresis + MALDI-TOF (PMF)	5	4
Martínez- Heredia et al., 2008 (24)	Progressive motility < 25% or total motility < 50% *	3–7 days abstinence 1-layer Percoll gradient	10	20	2D electrophoresis + MALDI-TOF/TOF	8	6
Siva et al., 2010 (25)	Progressive motility < 25% *	>3 days abstinence 2-layer Percoll gradient	20	17	2D electrophoresis + MALDI-TOF/TOF	3	4
Shen et al., 2013 (26)	Total motility < 40%**	3–7 days abstinence 1-layer Percoll gradient	30	30	2D electrophoresis + MALDI-TOF/TOF	3	10
Amaral et al., 2014b (27)	Progressive motility < 32%**	3–5 days abstinence 2-layer Percoll gradient	5	5	LC-MS/MS	30	50
Hashemitabar et al. 2015 (28)	Progressive motility ≤ 30% **	3–5 days abstinence 1-layer Percoll gradient	33	35	2D electrophoresis + MALDI-TOF/TOF	3	11
Saraswat et al., 2017 (29)	Total motility < 40%**	centrifugation	5	8	UPLC-MS/MS	164	172
Nowicka-Bauer et al., 2018 (30)	Total motility < 40%**	3–5 days abstinence 1-layer Percoll gradient	10	4	2D electrophoresis + MALDI-TOF (PMF)	7	16
Sinha et al., 2019 (31)	Progressive motility ≤ 32%**	centrifugation	70	70	2D-DIGE + MALDI-TOF/TOF	0	7
Guo et al., 2019 (32)	Progressive motility < 25% or total motility < 50%*	1-layer Percoll gradient	5	5	LC-MS/MS	83	67
Moscatelli et al., 2019 (33)	Progressive motility: ≤ 30%**	centrifugation	4	8	LC-MS/MS	67	79
Liang et al., 2021 (34)	Oligoasthenoteratozoospermia **	>3 days abstinence SpermGrad™ density gradient	12	12	LC-MS/MS	226	711
Yang et al., 2022 (35)	Progressive motility ≤ 32%**	3–7 days abstinence 1-layer Percoll gradient	7	11	LC-MS/MS	701	728
Becker et al., 2023 (36)	Asthenozoospermia or Oligoasthenoteratozoospermia **	>3 days abstinence PureSperm density gradient	31	22	LC-MS/MS	34	32
Chhikara et al., 2023 (37)	Total motility < 40%**	2-layer Percoll gradient	3	12	LC-MS/MS	7	56
Mirshahvaladi et al., 2023 (38)	Progressive motility ≤ 32% or total motility < 40%**	3–5 days abstinence, 1-layer Percoll gradient	40	40	LC-MS/MS	253	155

AZS, asthenozoospermic; DEPs, differentially expressed proteins; DIGE, differential gel electrophoresis; DR, downregulated; LC-MS/MS, liquid chromatography tandem mass spectrometry; MALDI-TOF, matrix-assisted laser desorption/ionization time of flight; NZS, normozoospermic; PMF, peptide mass fingerprinting; UPLC-MS/MS, Ultra-performance liquid chromatography-tandem mass spectrometry; UR, upregulated. *^15^; **^2^.

To increase robustness, we applied a recurrence cutoff whereby only DEPs consistently reported as either UR or DR in at least 2 independent studies were retained for initial global analysis. Using this criterion, 722 proteins were identified: 259 as UR, 442 as DR, and 21 reported in at least 2 studies for each direction of regulation (**Figure 2B**; **Supplementary Table 2**). Proteins in the latter group were excluded, resulting in a final dataset of 701 consistently recurrent DEPs, representing 28.5% of the initial non-redundant list.

### Recurrent UR proteins in AZS relative to NZS

**Figure 3:**
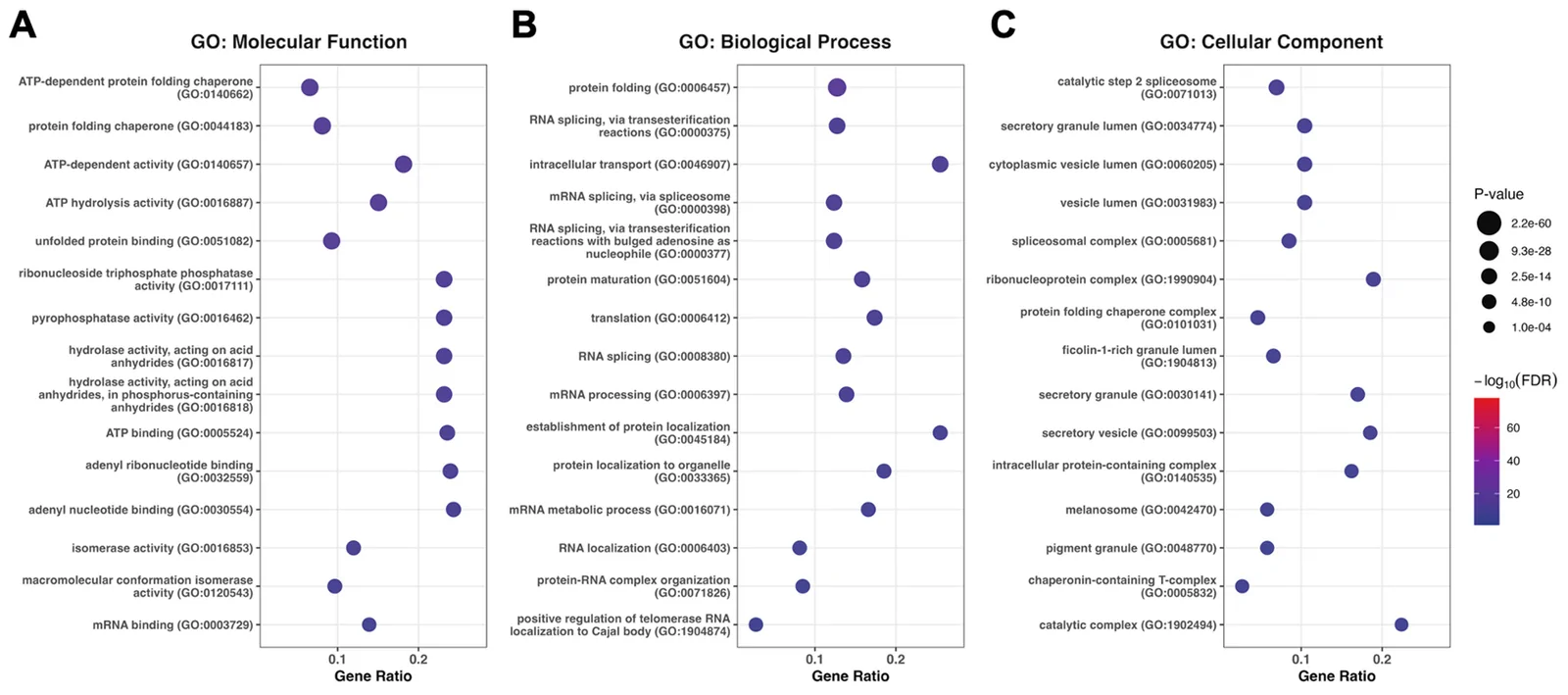
Functional enrichment analysis of recurrently upregulated sperm proteins in AZS relative to NZS. Dot plots showing significantly enriched Gene Ontology (GO) terms for molecular function **(A)**, biological process **(B)**, and cellular component **(C)** among recurrently upregulated proteins. The top 15 enriched GO terms ranked by FDR-adjusted *p*-value are shown. GeneRatio indicates the proportion of input proteins associated with each GO term.

The 259 recurrent UR proteins (20.7% of all UR proteins identified in the included studies) were subjected to GO functional enrichment analysis. At the molecular function level, terms related to ATP-dependent protein folding, unfolded protein binding, and nucleotide binding/ATP hydrolysis were significantly enriched (**Figure 3A**). Several terms displayed high gene ratios (~20–25%), particularly those associated with nucleotide binding and chaperone activity. Biological processes were mainly related to protein folding and maturation, intracellular transport, and RNA processing, particularly mRNA splicing (**Figure 3B**). At the cellular component level, enriched terms localized mainly to vesicle-related compartments, chaperone complexes, and ribonucleoprotein assemblies (**Figure 3C**).

KEGG, Reactome and HPO enrichment analyses were also performed for these recurrent UR proteins. KEGG analysis revealed significant enrichment of pathways related to protein processing in the endoplasmic reticulum, spliceosome, nucleocytoplasmic transport, aminoacyl-tRNA biosynthesis, and proteasome function (**Supplementary Figure 2A**). Among these, protein processing in the endoplasmic reticulum showed the highest gene ratio. Reactome analysis showed a broader enrichment profile in pathways involved in protein metabolism, cellular responses to stress and stimuli, RNA metabolism, translation, and mRNA splicing-related processes (**Supplementary Figure 2B**). Several enriched pathways were also associated with viral infection and host–pathogen interaction modules, likely reflecting the overrepresentation of shared host cellular machinery involved in RNA processing, translation, and protein folding. Finally, no HPO term enrichment was detected for these UR proteins (data not shown).

**Figure 4:**
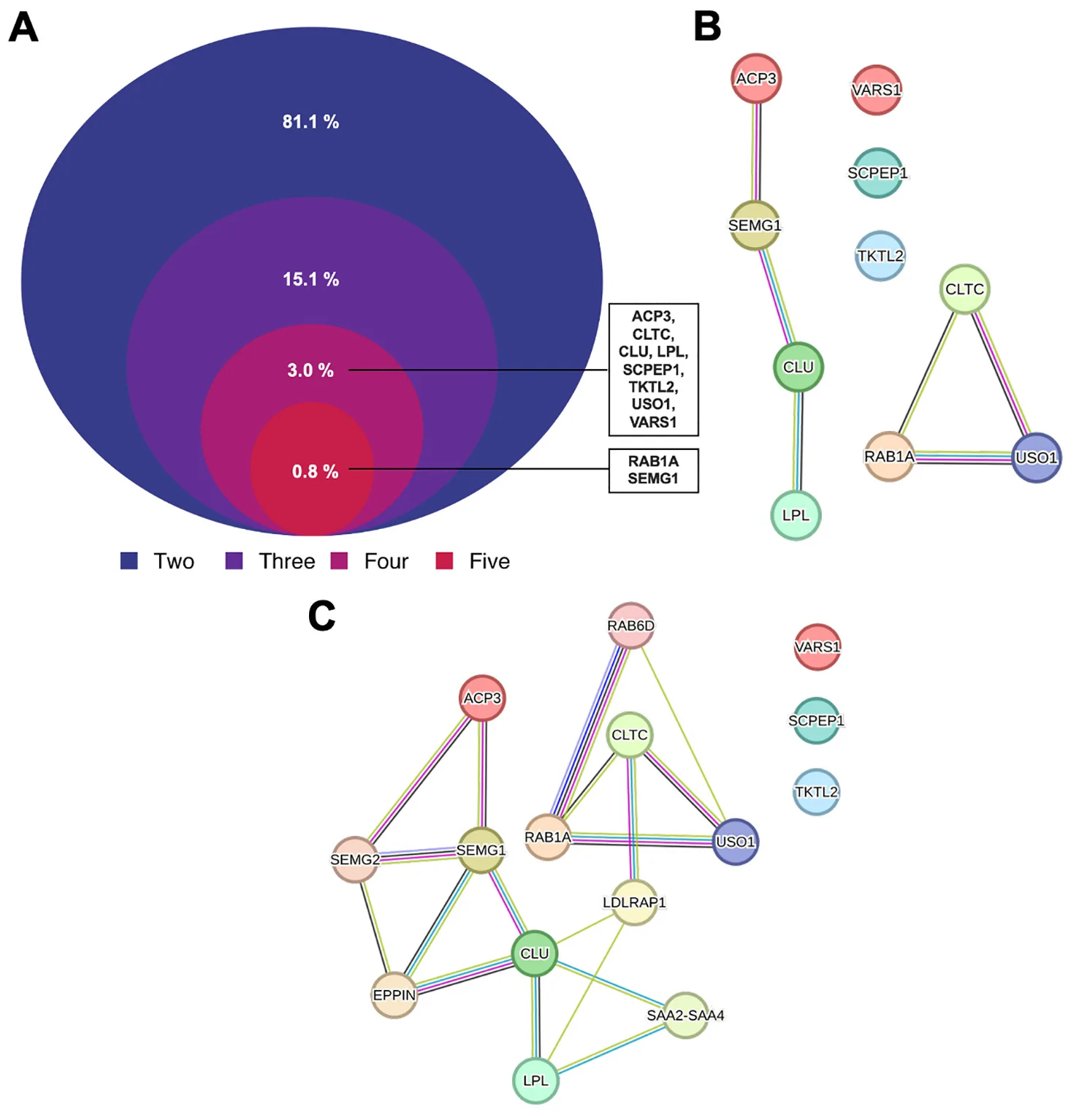
Recurrence and interaction networks of upregulated sperm proteins in AZS. **(A)** Euler diagram illustrating the set of upregulated (UR) proteins grouped according to the number of independent proteomic studies (2, 3, 4 or 5) in which they were reported in comparisons between AZS and NZS samples. **(B)** STRING protein–protein interaction network of the recurrent core UR proteins (reported in ≥ 4 studies). Edges represent known and predicted functional associations. **(C)** Expanded interaction network generated by adding first-shell interactors to the core network, revealing increased connectivity and additional interacting nodes.

Within this initial set of UR proteins, recurrence distribution was as follows: 210 (81.1%) were reported in 2 independent studies, 39 (15.1%) in 3 studies, 8 (3%) in 4 studies, and 2 (0.8%) in 5 studies (**Figure 4A**). Using this information, a more stringent recurrence threshold was applied for further analysis, by prioritizing DEPs with the highest study recurrence numbers. This core subset of highly recurrent proteins represented the strongest candidates for functional relevance. It comprised RAB1A and SEMG1 (detected in 5 independent studies) and ACP3, CLTC, CLU, LPL, SCPEP1, TKTL2, USO1, and VARS1 (detected in 4) (**Figure 4A**). To evaluate whether these core UR proteins were interconnected, STRING PPI analysis was carried out, revealing 2 major clusters with significant network enrichment (*p* = 7.86 × 10^-4^) (**Figure 4B**). One cluster included RAB1A, CLTC, and USO1, key regulators of vesicular trafficking and Golgi-to-ER transport. The second cluster was composed of SEMG1, ACP3, CLU, and LPL, proteins associated with seminal plasma composition, semen liquefaction, lipid metabolism, and protection against oxidative stress.

To further explore potential functional relationships, an expanded interaction network including predicted interactors was generated (**Figure 4C**), which showed increased connectivity (PPI enrichment *p* = 1.17 × 10^-4^). This network incorporated additional nodes, including RAB6D within the trafficking-related module and SAA2/SAA4 within the seminal plasma module. It also revealed a subnetwork involving SEMG1, SEMG2, and EPPIN, extracellular proteins previously implicated in the regulation of sperm motility (39). The two clusters were connected through predicted intermediates such as LDLRAP1. Although this particular protein was detected in a human sperm proteome (40), this association is supported mainly by STRING text-mining evidence and should be interpreted with caution in light of the spatial compartmentalization of the corresponding proteins within sperm.

Overall, the interaction network of the core UR proteins reflects and further refines the functional organization identified by the GO, KEGG, and Reactome enrichment analyses of the initial recurrent UR protein set. These observations place recurrent UR proteins within a limited number of functional domains involving proteostasis, intracellular trafficking, and extracellular seminal plasma factors.

### Recurrent DR proteins in AZS relative to NZS

**Figure 5:**
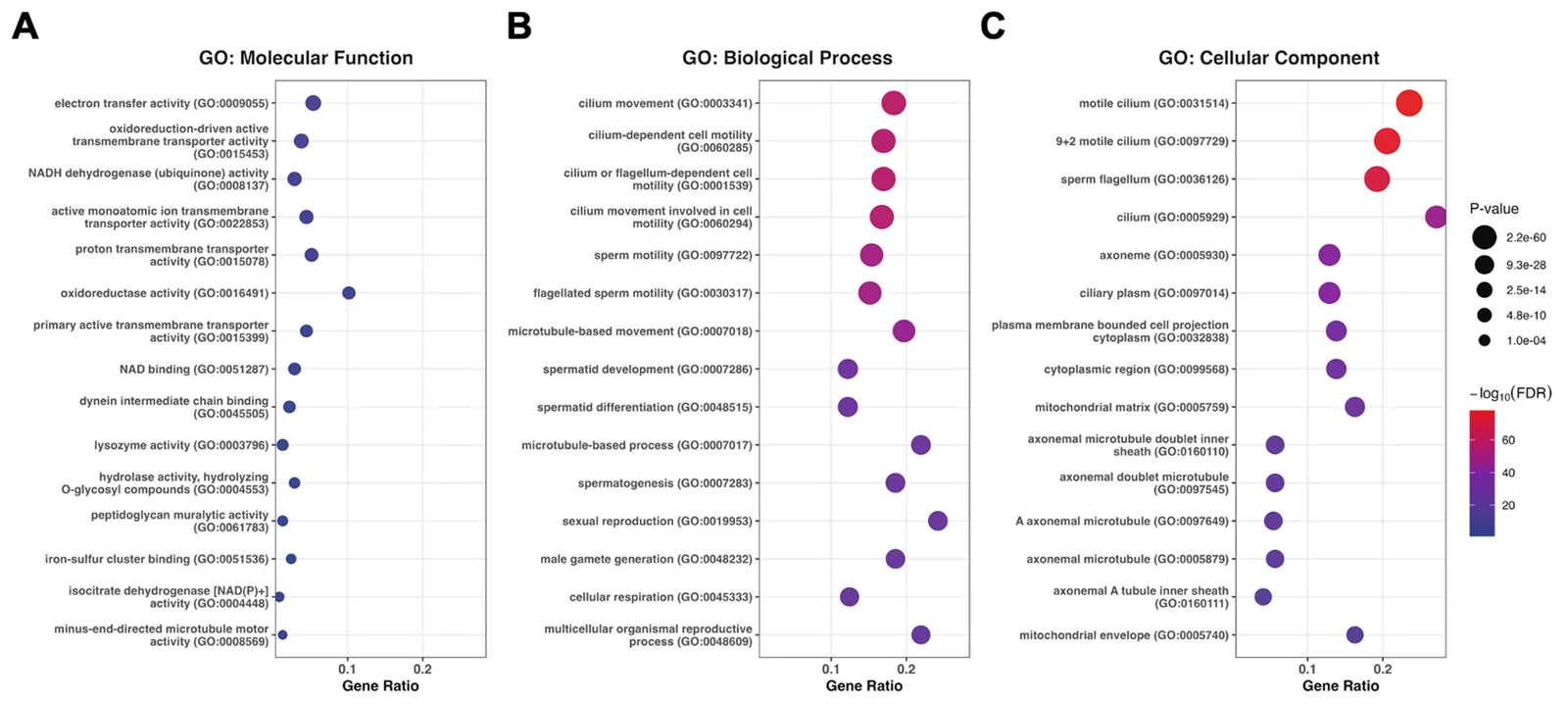
Functional enrichment analysis of recurrent downregulated sperm proteins in AZS relative to NZS. Dot plots showing significantly enriched Gene Ontology (GO) terms for molecular function **(A)**, biological process **(B)**, and cellular component **(C)** among recurrent downregulated proteins. The top 15 enriched GO terms ranked by FDR-adjusted *p*-value are shown. GeneRatio indicates the proportion of input proteins associated with each GO term.

The 442 recurrent DR proteins (30.6% of all DR proteins reported in the included studies) were subsequently subjected to enrichment analyses. According to GO analysis, molecular function terms were significantly enriched for electron transfer and oxidoreductase activities, particularly those associated with NAD(P)H-dependent reactions, ubiquinone-linked dehydrogenase functions, and redox-driven transmembrane transport processes (**Figure 5A**). Biological process enrichment was characterized by pathways associated with cilium- and flagellum-dependent cell motility, including microtubule-based movement and sperm motility, as well as processes linked to spermatogenesis, such as spermatid development and differentiation (**Figure 5B**). At the cellular component level, enriched terms primarily corresponded to motile cilium and axonemal structures, including the sperm flagellum, alongside mitochondrial compartments such as the mitochondrial matrix (**Figure 5C**).

KEGG pathway analysis of recurrent DR proteins revealed a predominance of metabolic pathways, including carbon metabolism, oxidative phosphorylation, non-alcoholic fatty liver disease, thermogenesis, and the citrate cycle (TCA cycle) (**Supplementary Figure 3A**), as well as pathways associated with neurodegenerative diseases (e.g., amyotrophic lateral sclerosis and Huntington disease). Reactome analysis similarly highlighted aerobic respiration and respiratory electron transport as the top enriched pathway, followed by mitochondrial protein degradation, respiratory electron transport, protein localization, Complex I biogenesis, among others (**Supplementary Figure 3B**). HPO enrichment identified top terms related to abnormality of acid-base homeostasis, abnormality of the mitochondrion, decreased fertility in males, abnormal sperm motility (**Supplementary Figure 3C**).

**Figure 6:**
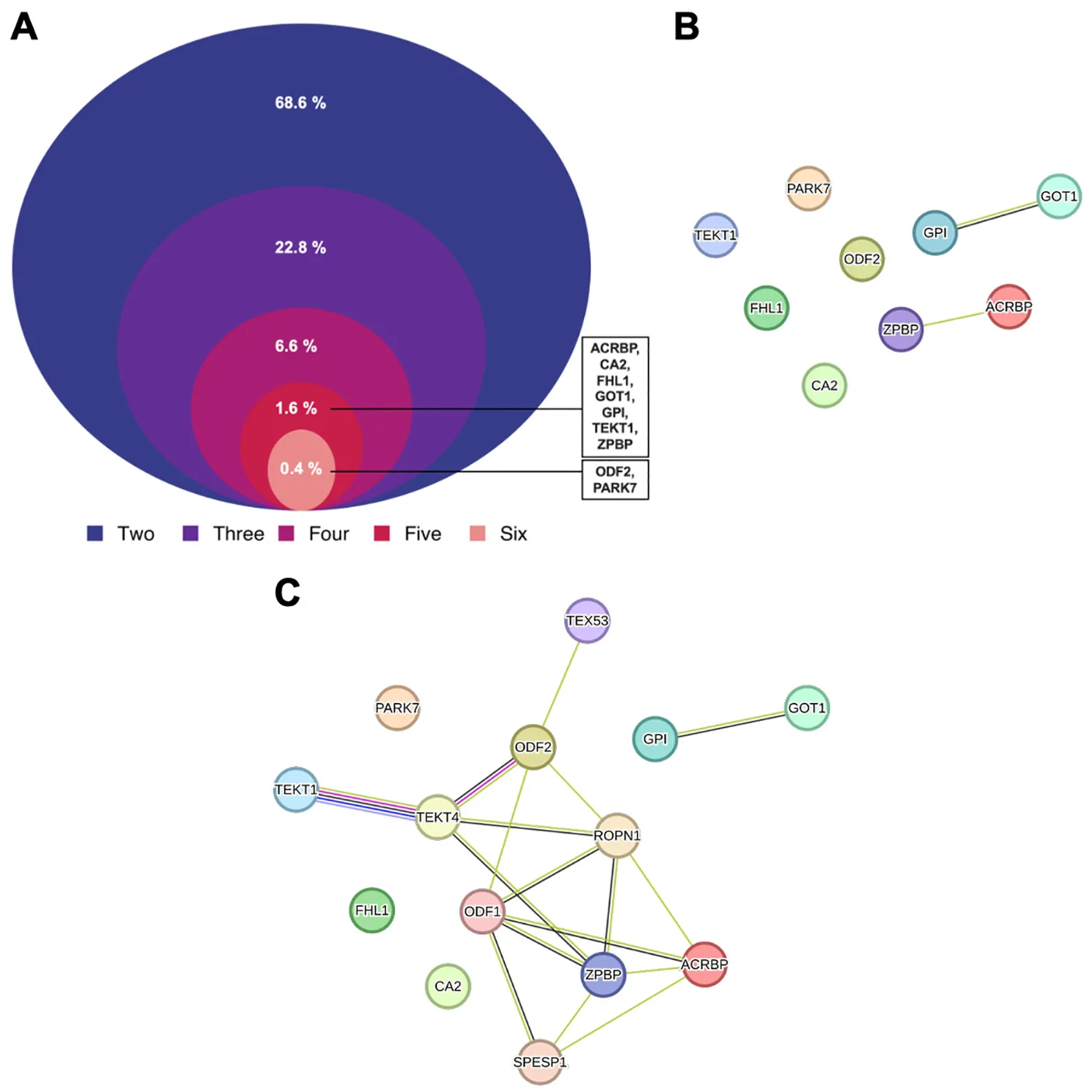
Recurrence and interaction networks of downregulated sperm proteins in AZS. **(A)** Euler diagram illustrating the set of downregulated (DR) proteins grouped according to the number of independent proteomic studies (2, 3, 4, 5 or 6) in which they were reported in comparisons between AZS and NZS samples. **(B)** STRING protein–protein interaction network of the recurrent core DR proteins (reported in ≥ 5 studies). Edges represent known and predicted functional associations. **(C)** Expanded interaction network generated by adding first-shell interactors to the core network, revealing increased connectivity and additional interacting nodes.

Within this protein group, 303 (68.6%) were reported in 2 independent studies, 101 (22.8%) in 3 studies, 29 (6.6%) in 4 studies, 7 (1.6%) in 5 studies, and 2 (0.4%) in 6 studies (**Figure 6A**). Applying the more stringent recurrence threshold, core DR proteins included PARK7 and ODF2 (6 studies) and ACRBP, CA2, FHL1, GOT1, GPI, TEKT1, and ZPBP (5 studies) (**Figure 6A**). STRING network analysis restricted to these proteins revealed a small interaction module centered on GPI and GOT1, two enzymes involved in intermediary metabolism, with limited overall connectivity (PPI enrichment *p* = 7.66 × 10^-2^) (**Figure 6B**). In the expanded interaction network (PPI enrichment *p* = 8.5 × 10^-6^), a central interaction module emerged comprising several proteins associated with sperm structural components, including ODF2, ODF1, TEKT4, ROPN1, ZPBP, and ACRBP (**Figure 6C**). Additional proteins such as PARK7, CA2, and FHL1 were located at the periphery of the network with limited connectivity with the main cluster.

Together, these interaction patterns place core DR proteins within functional networks associated with mitochondrial energy metabolism and flagellar structural organization, consistent with pathway and phenotype enrichments observed in the broader DR protein set.

### Integrated functional network of core proteins in AZS

**Figure 7:**
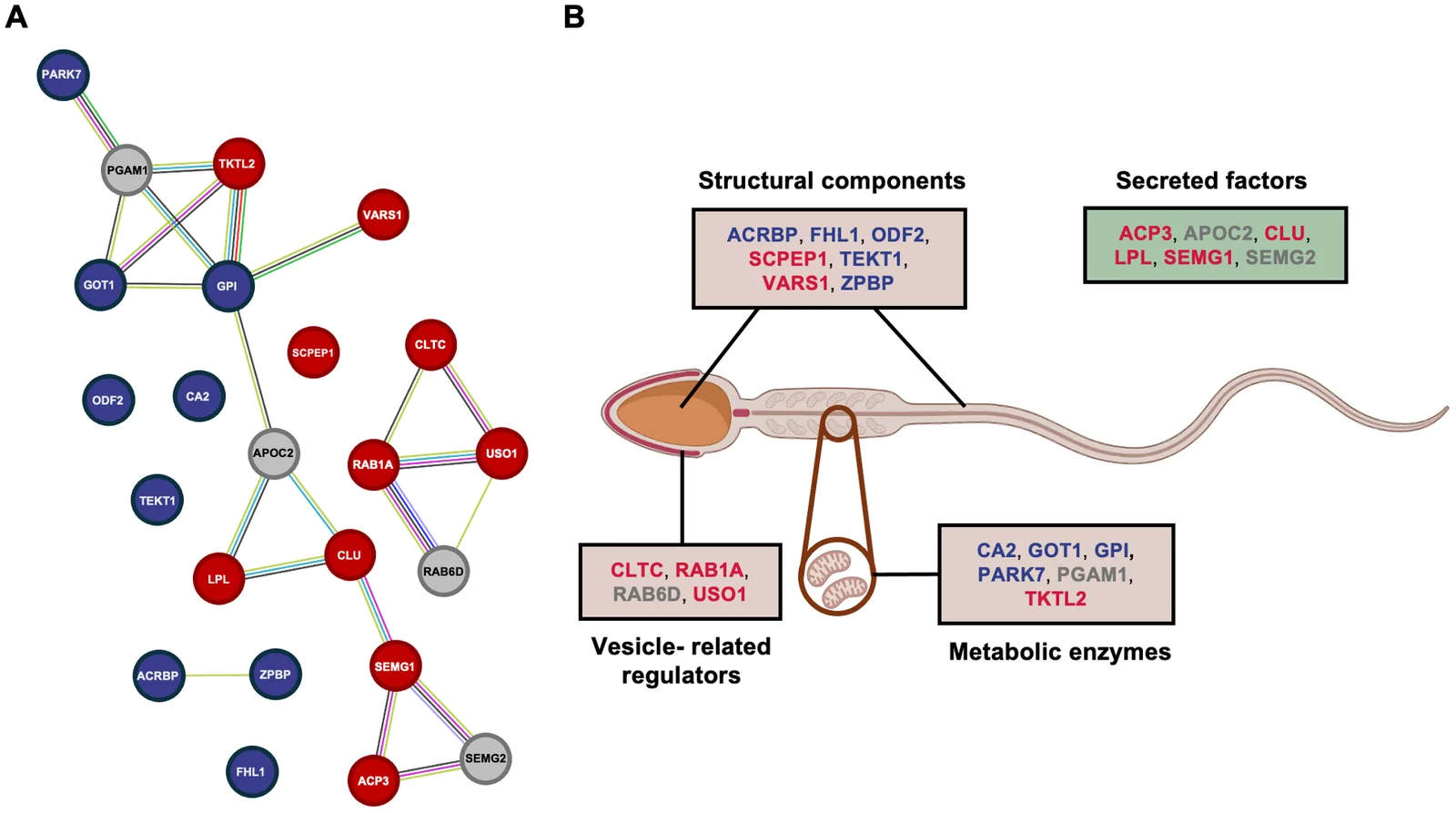
Integrated network of core proteins delineating functional modules as potential components of an AZS biomarker panel. **(A)** STRING network of the pooled core upregulated (UR) and downregulated (DR) proteins, showing four main clusters: structural, metabolic, vesicular trafficking, and extracellular/seminal. Nodes are colored by regulation status (UR, red; DR, blue; additional interactors, gray). Edge colors indicate interaction evidence: curated databases (light blue), experimental data (pink), gene neighborhood (green), gene fusions (red), gene co-occurrence (dark blue), text mining (yellow), co-expression (black), and protein homology (light purple). **(B)** Predicted subcellular localization of proteins included in each module within the sperm head and flagellum. Colors indicate regulation status (UR, red; DR, blue; additional interactors, gray). Created with Bio.Render.com.

To move beyond the classification of individual DEPs, a combined STRING-based PPI network analysis was performed using the pooled core UR and DR proteins to assess their collective organization (**Figure 7A**). This analysis revealed that the network naturally segregates into distinct clusters, reflecting groups of highly interconnected proteins. A metabolic cluster, centered on GPI, included GOT1, PGAM1, TKTL2 and PARK7, with peripheral CA2, enzymes predominantly associated with cytosolic and mitochondrial metabolic pathways, mainly localized in the sperm midpiece. A vesicular trafficking cluster comprised RAB1A, USO1, CLTC, and RAB6D, localized to acrosomal or vesicle-related compartments. A seminal plasma/extracellular cluster contained SEMG1, SEMG2, ACP3, CLU, LPL, and APOC2, secreted factors linked to the sperm surface. Additional proteins, including TEKT1, ODF2, FHL1, VARS1, SCPEP1, ZPBP, and ACRBP, showed limited connectivity and were positioned at the network periphery, corresponding mainly to structural components of the sperm flagellum or head.

Taken together, these clusters define an integrated functional network that, when considered in the broader biological context, reveals functional modules in distinct sperm compartments in AZS (**Figure 7B** and **Table 2**).

**Table 2:** Asthenozoospermia biomarker panel.

Acronym	Protein name	UniProtKB	NCBI gene ID	Study frequency	Direction of regulation	References
Vesicle- related regulatorsequation						
CLTC	clathrin heavy chain	Q00610	1213	4	UR	(29, 34, 35, 38)
RAB1A	Ras-related protein, Rab-1A	P62820	5861	5	UR	(29, 32, 34, 35, 38)
USO1	USO1 vesicle transport factor	O60763	8615	4	UR	(32, 34, 35, 38)
RAB6D	Ras-related protein Rab-6D	Q53S08	150786	–	INTERACTOME	–
Metabolic enzymes						
TKTL2	transketolase like 2	Q9H0I9	84076	4	UR	(29, 32, 35, 38)
CA2	carbonic anhydrase 2	P00918	760	5	DR	(23, 32, 34, 35, 37)
GOT1	glutamic-oxaloacetic transaminase 1	P17174	2805	5	DR	(32, 33, 34, 35, 36)
GPI	glucose-6-phosphate isomerase	P06744	2821	5	DR	(27, 32, 33, 35, 36)
PARK7	Parkinsonism associated deglycase	Q99497	11315	6	DR	(26, 29, 30, 32, 34, 37)
PGAM1	phosphoglycerate mutase 1	P18669	5223	1	DR/INTERACTOME	(32)
Structural components						
SCPEP1	serine carboxypeptidase 1	Q9HB40	59342	4	UR	(32, 33, 35, 36)
VARS1	valyl-tRNA synthetase 1	P26640	7407	4	UR	(29, 32, 35, 38)
ACRBP	acrosin binding protein	Q8NEB7	84519	5	DR	(29, 32, 34, 35, 37)
FHL1	four and a half LIM domains 1	Q13642	2273	5	DR	(27, 29, 34, 35, 36)
ODF2	outer dense fiber of sperm tails 2	Q5BJF6	4957	6	DR	(26, 28, 29, 30, 35, 38)
TEKT1	tektin 1	Q969V4	83659	5	DR	(25, 27, 30, 34, 38)
ZPBP	zona pellucida binding protein	Q9BS86	11055	5	DR	(32, 34, 35, 37, 38)
Secreted factors						
ACP3	acid phosphatase 3	P15309	55	4	UR	(27, 29, 32, 35)
CLU	clusterin	P10909	1191	4	UR	(24, 28, 32, 34)
LPL	lipoprotein lipase	P06858	4023	4	UR	(32, 35, 36, 38)
SEMG1	semenogelin 1	P04279	6406	5	UR	(24, 32, 33, 35, 36)
SEMG2	semenogelin 2	Q02383	6407	2	UR/INTERACTOME	(32, 36)
APOC2	apolipoprotein C-II	P02655	344	–	INTERACTOME	–

UR, upregulated; DR, downregulated.

## Discussion

In this study, we integrated independent human sperm proteomic datasets to identify robust molecular alterations associated with AZS. By applying a recurrence-based strategy and pathway enrichment analyses, a smaller core of UR and DR proteins emerged from the large number of DEPs reported across studies. Notably, rather than remaining as isolated protein signatures, these highly recurrent core DEPs define four major functional modules involving vesicle-mediated trafficking/proteostasis, mitochondrial metabolism, structural organization, and extracellular seminal plasma-associated factors, distributed across multiple sperm compartments. This modular architecture organizes previously fragmented protein lists into a cohesive model of impaired sperm motility, reflecting patterns of dysfunction among AZS patients. This aligns with the new conceptual “roadmap” for reproductive proteomics which advocates for integrative biological models over purely descriptive catalogs (14).

The vesicle-mediated trafficking/proteostasis functional module is centered on proteins involved in intracellular vesicle transport, including RAB1A, USO1, CLTC, and RAB6D. These proteins suggest disruption of trafficking pathways essential for acrosomal function and sperm compartmentalization. Although the core module itself was defined by trafficking-related proteins, it emerged from a broader recurrent UR protein signature enriched in protein folding, chaperone activity, RNA-associated processes, and other proteostasis-related pathways. Together, these observations support a link between altered intracellular trafficking and proteostasis imbalance, a process frequently associated with oxidative stress in male infertility (1, 41). While the association between proteostasis- and protein folding–related mechanisms and AZS is not novel, our integrative analysis broadens the etiological focus of this disease, highlighting this axis as a relevant component of its coordinated pathophysiological landscape and opening new avenues for mechanistic and diagnostic exploration.

The mitochondrial metabolism functional module includes recurrent dysregulation of enzymes involved in oxidative phosphorylation and intermediary metabolism, such as GPI, TKTL2, PARK7, and CA2, indicating impaired energy production and metabolic imbalance. This pattern is consistent with prior evidence linking reduced sperm motility to altered bioenergetic efficiency (30, 33, 42), as well as metabolomic studies suggesting compensatory metabolic remodeling (43). In addition, studies showing that dysregulation in fatty acid metabolism (44, 45) and iron-dependent ferroptosis (46) is associated with AZS further support the relevance of this metabolic module, where PARK7 may act as a critical mediator of redox homeostasis, as previously proposed (11, 30). The third functional module comprises structural proteins predominantly associated with the flagellar and head architecture, including TEKT1, ODF2, FHL1, VARS1, SCPEP1, ZPBP, and ACRBP, confirming the importance of cytoskeletal and flagellar organization as a coordinated structural feature of sperm motility. Altogether, the emergence of these two modules as key determinants of AZS validates the rationale underlying the strategy used in the present study.

The fourth functional module encompasses extracellular and seminal plasma-associated proteins such as SEMG1, SEMG2, CLU, ACP3, LPL, and APOC2, which are dynamically associated with the sperm surface through seminal plasma, extracellular vesicles, or epididymal transit. Many of these factors have been implicated in protein stabilization and protection against oxidative damage (47–49), suggesting that AZS-related alterations extend beyond intrinsic sperm defects to include dysregulation of the extracellular environment that modulates sperm function.

The biological relevance of the proposed modules is further supported by independent experimental evidence of several core DEPs (**Supplementary Table 3**). However, within this modular framework, we propose that its biological significance lies in the dysregulation of the functional modules themselves rather than in the specific proteins identified, since distinct protein alterations may converge on the same disrupted processes. As new proteomic datasets will accumulate over time, recurrent identification of any protein within these four modules would further validate the proposed architecture. Consistently with this view, the direction of regulation of proteins within the modules may also be less deterministic than traditionally assumed. In this regard, the excluded 21 recurrent proteins with inconsistent UR/DR directionality across studies may reflect dynamic regulation driven by post-translational modifications or context-dependent adaptive responses, as previously proposed (10). Supporting this concept, PDHB — one of the dual-assigned proteins identified here — interacts with PARK7, a recurrent core protein known to regulate PDHB phosphorylation and activity in other cellular systems (50). All these considerations, in the overall, highlight the complexity and heterogeneity of AZS etiology.

The nature of the proposed modules let us speculate the existence of functional interdependencies among them. Consequently, dysregulation of any one module could contribute, directly or indirectly, to alterations in the others. For example, dysregulated proteostasis and vesicle-mediated trafficking during spermatogenesis may represent upstream events, ultimately leading to the structural and metabolic alterations observed in the other modules. However, the available data, originally obtained from pooled sperm samples, do not allow us to determine whether these modules are simultaneously impaired in individual AZS patients, nor to establish the directionality of the relationships among them. Besides this, other limitations should be acknowledged, including the cross-sectional nature of the analyzed datasets and the technical heterogeneity inherent to proteomic studies performed across different platforms and sample preparation strategies. Nevertheless, the approach used to prioritize consistently reported proteins across independent cohorts mitigates these sources of variability. Finally, the proposed molecular framework requires validation in prospective and longitudinal cohorts.

Taken together, by applying recurrence-based filtering and network-based organization, we present a modular panel defined by four major functional modules, populated by recurrent core DEPs. Rather than representing a fixed set of disease-specific proteins, this framework suggests that AZS may arise from alterations affecting any of these functional modules, with different patients harboring distinct protein changes that converge on common biological processes. Importantly, this panel is not intended to replace conventional semen analysis, which remains a reliable tool for AZS diagnosis. Instead, these modules may provide a biologically informed basis for patient stratification according to the functional signature most prominently affected in each patient. This distinction could become clinically relevant in assisted reproduction, where the underlying molecular dysfunction may influence fertilization competence and early embryonic development even when ICSI is successfully used. Finally, the continuous generation of high-resolution proteomic data will enable the refinement of this perspective, facilitating the integration of emerging proteomic findings and supporting future multi-omics and AI-driven approaches for mechanism-informed diagnosis and personalized management of male infertility.

## Data Availability

Curated lists of differentially expressed proteins and results of integrative analyses supporting this study are included in the manuscript and supplementary materials; third party datasets are cited with references. Additional files, including analysis scripts and intermediate files, are available from the corresponding author upon reasonable request.
