# Supplementary material for: A modular, mechanism-informed biomarker panel for asthenozoospermia based on integrative sperm proteomics
Source: Front Endocrinol (Lausanne). 2026 Aug 10;17:1850544. doi: 10.3389/fendo.2026.1850544 (PMC13500254; doi:10.3389/fendo.2026.1850544)
Supplement: Supplementary file 1 [file Presentation1.pdf]

Supplementary Material

Supplementary Table 1. Studies excluded from the integrative proteomic analysis. Reference (first author and year), PMID, title, and exclusion criterion for each of the 200 studies retrieved in the literature search but not included in the analysis.

Supplementary Table 2. Recurrent differentially expressed proteins identified in sperm samples from AZS patients. Protein name, UniProt identifier, Acronym, NCBI Gene ID, study frequency, and direction of regulation (upregulated, UR; downregulated, DR; or inconsistently reported as both, UR/DR) for the 722 proteins recurrently reported as differentially expressed in 2 or more independent studies.

Supplementary Table 3. Experimental validation of core proteins. Acronym, experimental approach, concordance with the direction of regulation reported in Table 2, and reference (PMID) for core proteins with experimental or functional validation reported in the literature.

Supplementary Figure 1. Temporal distribution of differentially expressed proteins reported in AZS proteomic studies. Total number of significantly differentially expressed proteins (DEPs) identified in sperm samples from men with AZS, shown by year of publication.

Supplementary Figure 2. KEGG and Reactome enrichment analysis of recurrently upregulated sperm proteins in AZS relative to NZS. Dot plots showing the only 6 KEGG **(A)** and the top 15 Reactome **(B)** enriched pathways among recurrently upregulated proteins, ranked by FDR-adjusted *p*-value. GeneRatio indicates the proportion of input proteins associated with each pathway.

Supplementary Figure 3. KEGG, Reactome and HPO enrichment analysis of recurrent downregulated sperm proteins in AZS relative to NZS. Dot plots showing significantly enriched KEGG pathways **(A)**, Reactome pathways **(B)**, and Human Phenotype Ontology (HPO) terms **(C)** among recurrent downregulated proteins. The top 15 enriched terms ranked by FDR-adjusted *p*-value are shown. GeneRatio indicates the proportion of input proteins associated with each pathway or term.
