# Supplementary material for: A modular, mechanism-informed biomarker panel for asthenozoospermia based on integrative sperm proteomics
Source: Front Endocrinol (Lausanne). 2026 Aug 10;17:1850544. doi: 10.3389/fendo.2026.1850544 (PMC13500254; doi:10.3389/fendo.2026.1850544)
Supplement: Supplementary file 2 [file Table1.docx]

**Supplementary Table 1. Studies excluded from the integrative proteomic analysis.**

| **Study** | **PMID** | **Title** | **Exclusion criterion** |
| --- | --- | --- | --- |
| Bohring *et al.*, 2003 | 12721162 | Immune infertility: towards a better understanding of sperm (auto)-immunity | Review article |
| Lefièvre *et al.*, 2003 | 14753177 | Physiological and proteomic approaches to studying prefertilization events in the human | Review article |
| Domagala *et al.*, 2004 | 15035665 | Identification of sperm immunoreactive antigens for immunocontraceptive purposes: a review | Review article |
| Pilch *et al.*, 2006 | 16709260 | Large-scale and high-confidence proteomic analysis of human seminal plasma | Not a sperm proteomic analysis |
| Burden *et al.*, 2006 | 16373403 | Prostasomes--their effects on human male reproduction and fertility | Review article |
| Conner *et al.*, 2007 | 17566277 | Understanding the physiology of pre-fertilisation events in the human spermatozoa--a necessary prerequisite to developing rational therapy | Review article |
| Oliva *et al.*, 2007 | 17644990 | Proteomics of human spermatozoa, protamine content and assisted reproduction outcome | Review article |
| de Mateo *et al.*, 2007 | 18040982 | Marked correlations in protein expression identified by proteomic analysis of human spermatozoa | Not a direct AZS vs NZS comparison |
| Roy *et al.*, 2007 | 17244819 | Absence of tektin 4 causes asthenozoospermia and subfertility in male mice | Non-human study |
| Yamakawa *et al.*, 2007 | 17554110 | Comparative analysis of interindividual variations in the seminal plasma proteome of fertile men with identification of potential markers for azoospermia in infertile patients | Not a sperm proteomic analysis |
| Domagała *et al.*, 2007 | 17507387 | Application of proteomic methods for identification of sperm immunogenic antigens | Not a direct AZS vs NZS comparison |
| Karr, 2007 | 17911156 | Fruit flies and the sperm proteome | Non-human study |
| Huo *et al.*, 2008 | 18068167 | Identification of human spermatogenesis-related proteins by comparative proteomic analysis: a preliminary study | Not a sperm proteomic analysis |
| Oliva *et al.*, 2008 | 18543863 | Proteomics in the study of the sperm cell composition, differentiation and function | Review article |
| Aitken *et al.*, 2008 | 18179557 | The role of proteomics in understanding sperm cell biology | Review article |
| Barratt, 2008 | 18305001 | The human sperm proteome: the potential for new biomarkers of male fertility and a transformation in our understanding of the spermatozoon as a machine: commentary on the article 'Identification of proteomic differences in asthenozoospermic sperm samples' by Martinez *et al*. | Commentary article |
| Kriegel *et al.*, 2009 | 20021714 | Identification of diabetes- and obesity- associated proteomic changes in human spermatozoa by difference gel electrophoresis | Not a direct AZS vs NZS comparison |
| Baker *et al.*, 2009 | 19929613 | Proteomic insights into spermatozoa: critiques, comments and concerns | Review article |
| Oliva *et al.*, 2009 | 19212950 | Sperm cell proteomics | Review article |
| Wang *et al.*, 2009 | 19448646 | Proteomic analysis of seminal plasma from asthenozoospermia patients reveals proteins that affect oxidative stress responses and semen quality | Not a sperm proteomic analysis |
| Chan *et al.*, 2009 | 19678645 | Motility and protein phosphorylation in healthy and asthenozoospermic sperm | Not a global comparative proteomic analysis |
| Guo *et al.*, 2010 | 20099899 | Proteomic analysis of proteins involved in spermiogenesis in mouse | Non-human study |
| Ruwanpura *et al.*, 2010 | 20144980 | Hormonal regulation of male germ cell development | Review article |
| Tomar *et al.*, 2010 | 21133711 | Differential proteomics of sperm: insights, challenges and future prospects | Review article |
| Paasch *et al.*, 2011 | 21525168 | Identification of increased amounts of eppin protein complex components in sperm cells of diabetic and obese individuals by difference gel electrophoresis | Not a direct AZS vs NZS comparison |
| du Plessis *et al.*, 2011 | 21426553 | Proteomics: a subcellular look at spermatozoa | Review article |
| Domagala *et al.*, 2011 | 21470689 | An isoimmune response to human sperm clathrin in an infertile woman with systemic lupus erythematosus | Not a sperm proteomic analysis |
| Thacker *et al.*, 2011 | 21536282 | Evaluation of sperm proteins in infertile men: a proteomic approach | Sample preparation |
| Parte *et al.*, 2012 | 22796355 | Sperm phosphoproteome profiling by ultra- performance liquid chromatography followed by data independent analysis (LC-MS(E)) reveals altered proteomic signatures in asthenozoospermia | Not a global comparative proteomic analysis |
| Redgrove *et al.*, 2012 | 23209833 | The molecular chaperone HSPA2 plays a key role in regulating the expression of sperm surface receptors that mediate sperm-egg recognition | Not a direct AZS vs NZS comparison |
| Choi *et al.*, 2012 | 22819595 | Proteins associated with reproductive disorders in testes of human erythropoietin gene- harboring transgenic boars | Non-human study |
| Baker *et al.*, 2012 | 22788533 | Proteomic insights into the maturation and capacitation of mammalian spermatozoa | Review article |
| Dorus *et al.*, 2012 | 22788534 | Proteomic discovery of diverse immunity molecules in mammalian spermatozoa | Review article |
| Xu *et al.*, 2012 | 22771312 | Proteomic characteristics of spermatozoa in normozoospermic patients with infertility | Not a direct AZS vs NZS comparison |
| Fariello *et al.*, 2012 | 22863602 | Effect of smoking on the functional aspects of sperm and seminal plasma protein profiles in patients with varicocele | Not a direct AZS vs NZS comparison |
| Alvarez Sedó *et al.*, 2012 | 22511613 | Acrosomal biogenesis in human globozoospermia: immunocytochemical, ultrastructural and proteomic studies | Not a direct AZS vs NZS comparison |
| Govin *et al.*, 2012 | 21971310 | Proteomic strategy for the identification of critical actors in reorganization of the post-meiotic male genome | Not a direct AZS vs NZS comparison |
| Herwig *et al.*, 2013 | 23628110 | Proteomic analysis of seminal plasma from infertile patients with oligoasthenoteratozoospermia due to oxidative stress and comparison with fertile volunteers | Not a sperm proteomic analysis |
| Tasi *et al.*, 2013 | 23423614 | Characterization of 3-hydroxyisobutyrate dehydrogenase, HIBADH, as a sperm-motility marker | Not a global comparative proteomic analysis |
| Hosseinifar *et al.*, 2013 | 23374786 | Study of sperm protein profile in men with and without varicocele using two-dimensional gel electrophoresis | Not a direct AZS vs NZS comparison |
| Intasqui *et al.*, 2013 | 23893156 | Unraveling the sperm proteome and post-genomic pathways associated with sperm nuclear DNA fragmentation | Not a direct AZS vs NZS comparison |
| Hamada *et al.*, 2013 | 23312230 | Two-dimensional differential in-gel electrophoresis-based proteomics of male gametes in relation to oxidative stress | Not a direct AZS vs NZS comparison |
| Macleod *et al.*, 2013 | 23902417 | The application of proteomic approaches to the study of mammalian spermatogenesis and sperm function | Review article |
| Liu *et al.*, 2013 | 23436708 | Scanning of novel cancer/testis proteins by human testis proteomic analysis | Not a sperm proteomic analysis |
| Rolland *et al.*, 2013 | 23024119 | Identification of genital tract markers in the human seminal plasma using an integrative genomics approach | Not a sperm proteomic analysis |
| Sharma *et al.*, 2013 | 24004880 | Proteomic analysis of seminal fluid from men exhibiting oxidative stress | Not a sperm proteomic analysis |
| Sharma *et al.*, 2013 | 23688036 | Proteomic analysis of human spermatozoa proteins with oxidative stress | Not a direct AZS vs NZS comparison |
| Vazquez-Levin, 2013 | 23380187 | Proteomic analysis and sperm physiopathology: the two- dimensional difference in gel electrophoresis approach | Commentary article |
| Brázdová *et al.*, 2013 | 24280142 | Immunodominant semen proteins II: contribution of seminal proteins to female immune infertility | Not a sperm proteomic analysis |
| Drabovich *et al.*, 2013 | 24259048 | Differential diagnosis of azoospermia with proteomic biomarkers ECM1 and TEX101 quantified in seminal plasma | Not a sperm proteomic analysis |
| Zhu *et al.*, 2013 | 24115602 | Differential proteomic profiling in human spermatozoa that did or did not result in pregnancy via IVF and AID | Not a direct AZS vs NZS comparison |
| da Silva *et al.*, 2013 | 23849843 | Differential seminal plasma proteome according to semen retrieval in men with spinal cord injury | Not a sperm proteomic analysis |
| Sharma *et al.*, 2013 | 23663294 | Functional proteomic analysis of seminal plasma proteins in men with various semen parameters | Not a sperm proteomic analysis |
| Majumdar *et al.*, 2013 | 23375691 | Genomic and post-genomic leads toward regulation of spermatogenesis | Review article |
| Freour *et al.*, 2013 | 23427166 | Comparative proteomic analysis coupled with conventional protein assay as a strategy to identify predictors of successful testicular sperm extraction in patients with non-obstructive azoospermia | Not a direct AZS vs NZS comparison |
| Milardi *et al.*, 2013 | 23559416 | Proteomics of human seminal plasma: identification of biomarker candidates for fertility and infertility and the evolution of technology | Review article |
| Amaral *et al.*, 2014 | 24082039 | The combined human sperm proteome: cellular pathways and implications for basic and clinical science | Review article |
| Hosseinifar *et al.*, 2014 | 24760720 | Study of the effect of varicocelectomy on sperm proteins expression in patients with varicocele and poor sperm quality by using two-dimensional gel electrophoresis | Not a sperm proteomic analysis |
| Agarwal *et al.*, 2014 | 24813754 | Proteomics, oxidative stress and male infertility | Review article |
| Frapsauce *et al.*, 2014 | 24882558 | Proteomic identification of target proteins in normal but nonfertilizing sperm | Not a direct AZS vs NZS comparison |
| Pilatz *et al.*, 2014 | 24726218 | Acute epididymitis induces alterations in sperm protein composition | Patients with comorbidities |
| Hashemitabar *et al.*, 2014 | 25268621 | A proteomic analysis of human follicular fluid: comparison between younger and older women with normal FSH levels | Not a sperm proteomic analysis |
| McReynolds *et al.*, 2014 | 24934493 | Toward the identification of a subset of unexplained infertility: a sperm proteomic approach | Not a direct AZS vs NZS comparison |
| Giacomini *et al.*, 2015 | 25779018 | Comparative analysis of the seminal plasma proteomes of oligoasthenozoospermic and normozoospermic men | Not a sperm proteomic analysis |
| Lobo *et al.*, 2015 | 26618558 | Glucose Regulated Protein 78 Phosphorylation in Sperm Undergoes Dynamic Changes during Maturation | Not a global comparative proteomic analysis |
| Indu *et al.*, 2015 | 26432663 | Aberrant Expression of Dynein light chain 1 (DYNLT1) is Associated with Human Male Factor Infertility. | Not a global comparative proteomic analysis |
| Liu *et al.*, 2015 | 25637620 | Aged men share the sperm protein PATE1 defect with young asthenozoospermia patients | Not a global comparative proteomic analysis |
| Liu *et al.*, 2015 | 25293813 | Proteomic pattern changes associated with obesity-induced asthenozoospermia | Patients with comorbidities |
| Yang *et al.*, 2015 | 25476145 | Proteomic analysis of N-glycosylation of human seminal plasma | Not a sperm proteomic analysis |
| Holland *et al.*, 2015 | 24909132 | Comparative profiling of the sperm proteome | Review article |
| Agarwal *et al.*, 2015 | 25733269 | Differential proteomic profiling of spermatozoal proteins of infertile men with unilateral or bilateral varicocele | Not a sperm proteomic analysis |
| Castillo *et al.*, 2015 | 25926607 | Human sperm chromatin epigenetic potential: genomics, proteomics, and male infertility | Review article |
| Agarwal *et al.*, 2015 | 25890347 | Major protein alterations in spermatozoa from infertile men with unilateral varicocele | Not a sperm proteomic analysis |
| Mohanty *et al.*, 2015 | 25376881 | Sperm Proteome: What Is on the Horizon? | Review article |
| Agarwal *et al.*, 2016 | 25999357 | Spermatozoa protein alterations in infertile men with bilateral varicocele | Not a sperm proteomic analysis |
| Marques *et al.*, 2016 | 27827323 | Sequence variation at KLK and WFDC clusters and its association to semen hyperviscosity and other male infertility phenotypes | Not a global comparative proteomic analysis |
| Agarwal *et al.*, 2016 | 26732106 | Proteomic signatures of infertile men with clinical varicocele and their validation studies reveal mitochondrial dysfunction leading to infertility | Not a direct AZS vs NZS comparison |
| Hetherington *et al.*, 2016 | 27770032 | Deficiency in Outer Dense Fiber 1 Is a Marker and Potential Driver of Idiopathic Male Infertility | Not a direct AZS vs NZS comparison |
| Nowicka-Bauer *et al.*, 2016 | 26659478 | Proteomic identification of sperm antigens using serum samples from individuals with and without antisperm antibodies | Not a sperm proteomic analysis |
| Alikhani *et al.*, 2017 | 28213128 | Quantitative proteomic analysis of human testis reveals system-wide molecular and cellular pathways associated with non- obstructive azoospermia | Not a sperm proteomic analysis |
| Lin *et al.*, 2017 | 28067790 | RAB10 Interacts with the Male Germ Cell-Specific GTPase-Activating Protein during Mammalian Spermiogenesis | Not a direct AZS vs NZS comparison |
| Asghari *et al.*, 2017 | 28085499 | A sperm-specific proteome-scale metabolic network model identifies non-glycolytic genes for energy deficiency in asthenozoospermia | Not a global comparative proteomic analysis |
| Jodar *et al.*, 2017 | 27576136 | Semen proteomics and male infertility | Review article |
| Cao *et al.*, 2018 | 29482568 | Proteomic profile of human spermatozoa in healthy and asthenozoospermic individuals | Review article |
| Bracke *et al.*, 2018 | 29336995 | A search for molecular mechanisms underlying male idiopathic infertility | Review article |
| Liu *et al.*, 2018 | 29247744 | Disruption of Ssp411 causes impaired sperm head formation and male sterility in mice. | Non-human study |
| García-Rodríguez *et al.*, 2018 | 29726126 | Human prostasomes from normozoospermic and non-normozoospermic men show a differential protein expression pattern | Not a sperm proteomic analysis |
| Dipresa *et al.*, 2018 | 29667547 | New Markers for Predicting Fertility of the Male Gametes in the Post Genomic Era | Review article |
| Samanta *et al.*, 2018 | 29530785 | Proteomic Signatures of Sperm Mitochondria in Varicocele: Clinical Use as Biomarkers of Varicocele Associated Infertility | Not a direct AZS vs NZS comparison |
| Netherton *et al.*, 2018 | 29228106 | Proteomic analysis of good- and poor-quality human sperm demonstrates that several proteins are routinely aberrantly regulated | Not a direct AZS vs NZS comparison |
| Ayaz *et al.*, 2018 | 29656391 | Proteomic analysis of sperm proteins in infertile men with high levels of reactive oxygen species | Not a direct AZS vs NZS comparison |
| Mohanty *et al.*, 2018 | 30964084 | Redox regulation & sperm function: A proteomic insight | Review article |
| Samanta *et al.*, 2018 | 29704899 | The enigmatic seminal plasma: a proteomics insight from ejaculation to fertilization | Review article |
| Intasqui *et al.*, 2018 | 29205438 | Towards the identification of reliable sperm biomarkers for male infertility: A sperm proteomic approach | Not a direct AZS vs NZS comparison |
| Dias *et al.*, 2019 | 30626014 | Proteomic Signatures Reveal Differences in Stress Response, Antioxidant Defense and Proteasomal Activity in Fertile Men with High Seminal ROS Levels | Not a direct AZS vs NZS comparison |
| Panner Selvam *et al.*, 2019 | 30595021 | Molecular Pathways Associated with Sperm Biofunction Are Not Affected by the Presence of Round Cell and Leukocyte Proteins in Human Sperm Proteome | Not a sperm proteomic analysis |
| Shen *et al.*, 2019 | 30683861 | Loss-of- function mutations in QRICH2 cause male infertility with multiple morphological abnormalities of the sperm flagella | Not a direct AZS vs NZS comparison |
| Liu *et al.*, 2019 | 30867229 | Integrated Analyses of Phenotype and Quantitative Proteome of CMTM4 Deficient Mice Reveal Its Association with Male Fertility | Not a direct AZS vs NZS comparison |
| He *et al.*, 2019 | 30950109 | 14-3-3 epsilon plays an important role in testicular germ cell apoptosis: A functional proteomic study of experimental varicocele | Non-human study |
| Schiza *et al.*, 2019 | 30429210 | Identification of TEX101-associated Proteins Through Proteomic Measurement of Human Spermatozoa Homozygous for the Missense Variant rs35033974 | Not a direct AZS vs NZS comparison |
| Sha *et al.*, 2019 | 30298696 | Biallelic mutations in PMFBP1 cause acephalic spermatozoa | Not a direct AZS vs NZS comparison |
| Camargo *et al.*, 2019 | 30710756 | Molecular pathways of varicocele and its repair - A paired labelled shotgun proteomics approach | Not a direct AZS vs NZS comparison |
| Alfano *et al.*, 2019 | 30929729 | Impaired testicular signaling of vitamin A and vitamin K contributes to the aberrant composition of the extracellular matrix in idiopathic germ cell aplasia | Not a sperm proteomic analysis |
| Ferrero *et al.*, 2019 | 31642202 | Proteomic Research of Molecular Defects in Globozoospermia | Not a direct AZS vs NZS comparison |
| Panner Selvam *et al.*, 2019 | 30924599 | A quantitative global proteomics approach to understanding the functional pathways dysregulated in the spermatozoa of asthenozoospermic testicular cancer patients | Patients with comorbidities |
| Lin *et al.*, 2019 | 31215738 | Proteomic analysis of seminal extracellular vesicle proteins involved in asthenozoospermia by iTRAQ | Not a sperm proteomic analysis |
| Murdica *et al.*, 2019 | 31355853 | Proteomic analysis reveals the negative modulator of sperm function glycodelin as over- represented in semen exosomes isolated from asthenozoospermic patients | Not a sperm proteomic analysis |
| Wu *et al.*, 2019 | 30984777 | Quantitative Proteomic Analysis of Human Seminal Plasma from Normozoospermic and Asthenozoospermic Individuals | Not a sperm proteomic analysis |
| Peng *et al.*, 2019 | 31651332 | Proteomic alterations underlie an association with teratozoospermia in obese mice sperm | Non-human study |
| Samanta *et al.*, 2019 | 30381577 | Proteomic analysis reveals dysregulated cell signaling in ejaculated spermatozoa from infertile men | Not a direct AZS vs NZS comparison |
| Agarwal *et al.*, 2019 | 31056976 | Sperm DNA damage and its impact on male reproductive health: a critical review for clinicians, reproductive professionals and researchers | Review article |
| Panner Selvam *et al.*, 2019 | 31336797 | Sperm Proteome Analysis and Identification of Fertility-Associated Biomarkers in Unexplained Male Infertility | Not a direct AZS vs NZS comparison |
| Guo *et al.*, 2019 | 31424156 | Proteomic Analysis of Dpy19l2-Deficient Human Globozoospermia Reveals Multiple Molecular Defects | Not a direct AZS vs NZS comparison |
| Panner Selvam *et al.*, 2019 | 30764484 | Altered Molecular Pathways in the Proteome of Cryopreserved Sperm in Testicular Cancer Patients before Treatment | Patients with comorbidities |
| Barrachina *et al.*, 2019 | 30518674 | Stable-protein Pair Analysis as A Novel Strategy to Identify Proteomic Signatures: Application To Seminal Plasma From Infertile Patients | Not a sperm proteomic analysis |
| Agarwal *et al.*, 2020 | 32120839 | Proteomic Analyses of Human Sperm Cells: Understanding the Role of Proteins and Molecular Pathways Affecting Male Reproductive Health | Review article |
| Heidary *et al.*, 2020 | 30198353 | Genetic aspects of idiopathic asthenozoospermia as a cause of male infertility | Review article |
| Candenas *et al.*, 2020 | 32987677 | Exosome Composition and Seminal Plasma Proteome: A Promising Source of Biomarkers of Male Infertility. | Review article |
| Wang *et al.*, 2020 | 32976492 | Deficiency of the Tbc1d21 gene causes male infertility with morphological abnormalities of the sperm mitochondria and flagellum in mice | Not a direct AZS vs NZS comparison |
| Dougherty *et al.*, 2020 | 33139725 | CFAP45 deficiency causes situs abnormalities and asthenospermia by disrupting an axonemal adenine nucleotide homeostasis module. | Not a global comparative proteomic analysis |
| Agarwal *et al.*, 2020 | 31861964 | Unraveling the Footsteps of Proteomics in Male Reproductive Research: A Scientometric Approach. Antioxid Redox Signal | Review article |
| Martin-Hidalgo *et al.*, 2020 | 31972345 | Human sperm phosphoproteome reveals differential phosphoprotein signatures that regulate human sperm motility | Not a global comparative proteomic analysis |
| Velez *et al.*, 2020 | 33008501 | Personalized Medicine for the Infertile Male | Review article |
| Cannarella *et al.*, 2020 | 33266209 | Seminal Plasma Proteomic Biomarkers of Oxidative Stress | Not a sperm proteomic analysis |
| Jodar *et al.*, 2020 | 32444165 | Sperm proteomic changes associated with early embryo quality after ICSI | Not a direct AZS vs NZS comparison |
| Cannarella *et al.*, 2020 | 33022946 | Seminal Plasma Transcriptome and Proteome: Towards a Molecular Approach in the Diagnosis of Idiopathic Male Infertility | Not a sperm proteomic analysis |
| Chan *et al.*, 2020 | 32198406 | Reproductive tract extracellular vesicles are sufficient to transmit intergenerational stress and program neurodevelopment | Not a sperm proteomic analysis |
| Fernandez-Encinas *et al.*, 2020 | 32708913 | Proteomic Analysis in Seminal Plasma of Fertile Donors and Infertile Patients with Sperm DNA Fragmentation | Not a sperm proteomic analysis |
| Netherton *et al.*, 2020 | 31848259 | Proteomic Analysis Reveals that Topoisomerase 2A is Associated with Defective Sperm Head Morphology | Not a direct AZS vs NZS comparison |
| Mayerhofer, 2020 | 31237067 | Peritubular cells of the human testis: prostaglandin E<sub>2</sub> and more | Review article |
| Wu *et al.*, 2020 | 32787870 | Effect of transient scrotal hyperthermia on human sperm: an iTRAQ-based proteomic analysis | Not a direct AZS vs NZS comparison |
| Panner Selvam *et al.*, 2020 | 32942548 | Dysregulation of Key Proteins Associated with Sperm Motility and Fertility Potential in Cancer Patients | Patients with comorbidities |
| Martins *et al.*, 2020 | 32372034 | Alterations in seminal plasma proteomic profile in men with primary and secondary infertility | Not a sperm proteomic analysis |
| Swain *et al.*, 2020 | 31691576 | Aberrant Upregulation of Compensatory Redox Molecular Machines May Contribute to Sperm Dysfunction in Infertile Men with Unilateral Varicocele: A Proteomic Insight | Not a direct AZS vs NZS comparison |
| Liu *et al.*, 2020 | 31364287 | Characterization of seminal plasma proteomic alterations associated with the IVF and rescue-ICSI pregnancy in assisted reproduction | Not a sperm proteomic analysis |
| Rivera-Egea *et al.*, 2021 | 33835370 | Differential sperm proteomic profiles according to pregnancy achievement in intracytoplasmic sperm injection cycles: a pilot study | Not a direct AZS vs NZS comparison |
| Naglot *et al.*, 2021 | 34329819 | Label-free proteomics of spermatozoa identifies candidate protein markers of idiopathic recurrent pregnancy loss | Not a direct AZS vs NZS comparison |
| Liang *et al*., 2021 | 33375868 | Comparison of proteomic profiles from the testicular tissue of males with impaired and normal spermatogenesis | Not a sperm proteomic analysis |
| Liu *et al.*, 2021 | 33980814 | Loss of CEP70 function affects acrosome biogenesis and flagella formation during spermiogenesis | Not a direct AZS vs NZS comparison |
| Chen *et al.*, 2021 | 34172998 | Loss of SPACA1 function causes autosomal recessive globozoospermia by damaging the acrosome-acroplaxome complex | Not a direct AZS vs NZS comparison |
| Ribeiro *et al.*, 2021 | 33567922 | Insights and clinical potential of proteomics in understanding spermatogenesis | Review article |
| Zarezadeh *et al.*, 2021 | 33860468 | Omics in Seminal Plasma: An Effective Strategy for Predicting Sperm Retrieval Outcome in Non-obstructive Azoospermia | Not a sperm proteomic analysis |
| Panner Selvam *et al.*, 2021 | 32453911 | Protein profiling in unlocking the basis of varicocele-associated infertility | Review article |
| Estudillo *et al.*, 2021 | 34639209 | Cryopreservation of Gametes and Embryos and Their Molecular Changes | Review article |
| Hua *et al.*, 2021 | 33095425 | MicroRNA-449a Suppresses Mouse Spermatogonia Proliferation via Inhibition of CEP55 | Not a direct AZS vs NZS comparison |
| Lei *et al.*, 2021 | 34428398 | Autophagic elimination of ribosomes during spermiogenesis provides energy for flagellar motility | Non-human study |
| Torra-Massana *et al.*, 2021 | 33615715 | Altered mitochondrial function in spermatozoa from patients with repetitive fertilization failure after ICSI revealed by proteomics | Not a direct AZS vs NZS comparison |
| Teke *et al.*, 2021 | 34009669 | SERPIN A5 may have a potential as a biomarker in reflecting the improvement of semen quality in infertile men who underwent varicocele repair | Not a direct AZS vs NZS comparison |
| Qi *et al.*, 2021 | 34664184 | A novel posttranslational modification of histone, H3 S-sulfhydration, is down-regulated in asthenozoospermic sperm | Not a global comparative proteomic analysis |
| Chen *et al.*, 2021 | 33279973 | Testis developmental related gene 1 (TDRG1) encodes a progressive motility- associated protein in human spermatozoa | Not a global comparative proteomic analysis |
| Cheung *et al.*, 2021 | 33877510 | Single-center thorough evaluation and targeted treatment of globozoospermic men | Not a direct AZS vs NZS comparison |
| Ribeiro *et al.*, 2022 | 34847748 | Exploring the Role of Oxidative Stress in Sperm Motility: A Proteomic Network Approach. Antioxid Redox Signal | Review article |
| Tsai *et al.*, 2022 | 36295569 | Localization Patterns of RAB3C Are Associated with Murine and Human Sperm Formation | Not a direct AZS vs NZS comparison |
| Tan *et al.*, 2022 | 35366759 | Extracellular vesicles derived from human Sertoli cells: characterizations, proteomic analysis, and miRNA profiling. | Not a sperm proteomic analysis |
| Grassi *et al.*, 2022 | 35742939 | Targeted Analysis of HSP70 Isoforms in Human Spermatozoa in the Context of Capacitation and Motility. | Not a direct AZS vs NZS comparison |
| Voigt *et al.*, 2022 | 35856882 | Metabolic transitions define spermatogonial stem cell maturation | Not a sperm proteomic analysis |
| Sysoeva *et al.*, 2022 | 36094592 | Influence of Extracellular Vesicles from the Follicular Fluid of Young Women and Women of Advanced Maternal Age with Different miRNA Profiles on Sperm Functional Properties | Not a sperm proteomic analysis |
| Corda *et al.*, 2022 | 36235040 | G-Protein Coupled Receptors in Human Sperm: An In Silico Approach to Identify Potential Modulatory Targets | Not a direct AZS vs NZS comparison |
| Grande *et al.*, 2022 | 35663320 | The Role of Testosterone in Spermatogenesis: Lessons From Proteome Profiling of Human Spermatozoa in Testosterone Deficiency | Not a direct AZS vs NZS comparison |
| Zhang *et al.*, 2022 | 35368057 | Recent progress of proteomic analysis on spermatogenesis | Review article |
| Patankar *et al.*, 2022 | 35840050 | Proteomic and genetic dissection of testis-specific histone 2B in infertile men reveals its contribution to meiosis and sperm motility | Not a direct AZS vs NZS comparison |
| Li *et al.*, 2022 | 34755699 | Sperm flagellar 2 (SPEF2) is essential for sperm flagellar assembly in humans | Not a direct AZS vs NZS comparison |
| Panner Selvam *et al.*, 2022 | 35641866 | Molecular Interactions Associated with Oxidative Stress-Mediated Male Infertility: Sperm and Seminal Plasma Proteomics | Review article |
| Roy *et al.*, 2023 | 37033249 | Altered ureido protein modification profiles in seminal plasma extracellular vesicles of non-normozoospermic men | Not a sperm proteomic analysis |
| Hua *et al.*, 2023 | 37070736 | Homozygous frameshift variant in POC1B causes male infertility with oligoasthenoteratozoospermia in human and mice | Not a global comparative proteomic analysis |
| Zhang *et al.*, 2023 | 37667331 | Loss of ACTL7A causes small head sperm by defective acrosome-acroplaxome- manchette complex | Non-human study |
| Dacheux *et al.*, 2023 | 37934199 | Novel axonemal protein ZMYND12 interacts with TTC29 and DNAH1, and is required for male fertility and flagellum function | Not a global comparative proteomic analysis |
| Guo *et al.*, 2023 | 36686470 | A new perspective on semen quality of aged male: The characteristics of metabolomics and proteomics | Not a direct AZS vs NZS comparison |
| Hu *et al.*, 2023 | 37903275 | CAMSAP1 role in orchestrating structure and dynamics of manchette microtubule minus-ends impacts male fertility during spermiogenesis | Non-human study |
| Wu *et al.*, 2023 | 37050850 | Comparative proteomic analysis identifies differentially expressed proteins associated with meiotic arrest in cattle-yak hybrids | Non-human study |
| Yu *et al.*, 2023 | 36624393 | Comparative proteomic analysis of seminal plasma exosomes in buffalo with high and low sperm motility | Non-human study |
| Arslan *et al.*, 2023 | 37750643 | Comparison of sperm carnitine profiles of normospermic, oligospermic and azospermic individuals | Not a direct AZS vs NZS comparison |
| Castillo *et al.*, 2023 | 37632247 | Proteomics of human spermatozoa. | Review article |
| Lin *et al.*, 2023 | 37833433 | Low acrosin activity is associated with decreased Spam1/acrosin expression and GSH deficiency-caused premature acrosome release of human sperm cells | Not a direct AZS vs NZS comparison |
| Vallet-Buisan *et al.*, 2023 | 36882116 | Contribution of semen to early embryo development: fertilization and beyond | Review article |
| Pacheco *et al.*, 2023 | 37892144 | New Insights on Sperm Function in Male Infertility of Unknown Origin: A Multimodal Approach | Not a direct AZS vs NZS comparison |
| Nixon *et al.*, 2023 | 36909306 | New horizons in human sperm selection for assisted reproduction | Review article |
| Wen *et al.*, 2023 | 35973810 | Sequencing of the <i>ZMYND15</i> gene in a cohort of infertile Chinese men reveals novel mutations in patients with teratozoospermia | Not a direct AZS vs NZS comparison |
| Ali *et al.*, 2023 | 37209627 | Spectacular role of epididymis and bio- active cargo of nano-scale exosome in sperm maturation: A review | Review article |
| Greither *et al.*, 2023 | 36896575 | The human sperm proteome-Toward a panel for male fertility testing | Sample preparation |
| Sutton *et al.*, 2023 | 37452050 | Molecular dissection and testing of PRSS37 function through LC-MS/MS and the generation of a PRSS37 humanized mouse model | Non-human study |
| Xin *et al.*, 2024 | 39073901 | Site-specific N-glycoproteomic analysis reveals up-regulated fucosylation in seminal plasma of asthenozoospermia. | Not a sperm proteomic analysis |
| Schon *et al.*, 2024 | 38065301 | Proteomic analysis of human sperm reveals changes in protamine 1 phosphorylation in men with infertility. | Not a global comparative proteomic analysis |
| Tian *et al.*, 2024 | 38797435 | Global proteomic analyses of lysine acetylation, malonylation, succinylation, and crotonylation in human sperm reveal their involvement in male fertility. | Not a direct AZS vs NZS comparison |
| Mancini *et al.*, 2024 | 37480475 | Combined evaluation of prolactin-induced peptide (PIP) and extracellular signal-regulated kinase (ERK) as new sperm biomarkers of FSH treatment efficacy in normogonadotropic idiopathic infertile men. | Not a global comparative proteomic analysis |
| Shen *et al.*, 2024 | 38727211 | Seminal plasma S100A8/A9 as a potential biomarker of genital tract inflammation | Non-human study |
| Chen *et al.*, 2024 | 38168070 | STYXL1 regulates CCT complex assembly and flagellar tubulin folding in sperm formation | Non-human study |
| Fietz *et al.*, 2024 | 38654926 | Proteomic biomarkers in seminal plasma as predictors of reproductive potential in azoospermic men | Not a sperm proteomic analysis |
| Garcia *et al.*, 2024 | 39422082 | Novel Genes of the Male Reproductive System: Potential Roles in Male Reproduction and as Non-hormonal Male Contraceptive Targets | Review article |
| Hu *et al*., 2024 | 38605082 | Hypoxia enhances autophagy level of human sperms | Dataset not available |
| Wang *et al.*, 2024 | 39406274 | Investigating the effects of COVID-19 on sperm in male smokers: A prospective integrated proteomic and metabolomic study | Not a direct AZS vs NZS comparison |
| Parkes *et al.*, 2024 | 38536015 | Bringing proteomics to bear on male fertility: key lessons | Review article |
| Ji *et al.*, 2024 | 37607985 | A Proteomic Analysis of Human Follicular Fluid: Proteomic Profile Associated with Embryo Quality | Not a sperm proteomic analysis |
| Kaltsas *et al.*, 2024 | 39768802 | Paternal Contributions to Recurrent Pregnancy Loss: Mechanisms, Biomarkers, and Therapeutic Approaches | Review article |
| Santiago *et al.*, 2025 | 40497989 | Advanced Paternal Age and Sperm Proteome Dynamics: A Possible Explanation for Age-Associated Male Fertility Decline | Not a direct AZS vs NZS comparison |
| Tang *et al.*, 2025 | 40009974 | Novel variants in DNAH17 cause sperm flagellar outer dynein arm defects but not total fertilization failure after ICSI | Not a direct AZS vs NZS comparison |
| Governini *et al.*, 2025 | 40563475 | Unraveling the Multi-Omic Landscape of Extracellular Vesicles in Human Seminal Plasma | Not a sperm proteomic analysis |
| Zhou *et al.*, 2025 | 39704931 | Homozygous deleterious variants in MYCBPAP induce asthenoteratozoospermia involving abnormal acrosome biogenesis, manchette structure and sperm tail assembly in humans and mice | Not a global comparative proteomic analysis |
| Shi *et al.*, 2025 | 38934611 | A novel homozygous LRRC6 mutation causes male infertility with asthenozoospermia and primary ciliary dyskinesia in humans | Not a global comparative proteomic analysis |
| Li *et al.*, 2025 | 39774634 | Impact of DNAH3 deficiency on sperm energy metabolism and motility leading to asthenozoospermia | Not a global comparative proteomic analysis |

AZS: asthenozoospermic, NZS: normozoospermic.
