# Supplementary material for: A modular, mechanism-informed biomarker panel for asthenozoospermia based on integrative sperm proteomics
Source: Front Endocrinol (Lausanne). 2026 Aug 10;17:1850544. doi: 10.3389/fendo.2026.1850544 (PMC13500254; doi:10.3389/fendo.2026.1850544)
Supplement: Supplementary file 3 [file Table3.docx]

**Supplementary Table 3. Experimental validation of core proteins**

| **Acronym** | **Experimental approach** | **Concordance with Table 2** | **Reference (PMID)** |
| --- | --- | --- | --- |
| ACRBP | Knock out mouse model | yes | 27303034 |
| CA2 | Knock out mouse model | yes | 26487715 |
| GPI | Western blot and ELISA (human) | yes | 31394311 |
| ODF2 | Whole exome sequencing/Immunofluorescence (human) | yes | 35102900 |
|  | Western blot (human), Knock out mouse model | yes | 29168316 |
|  | Western blot (buffalo) | yes | 25809445 |
| PARK7 | Western blot and ELISA (human) | yes | 21575935 |
|  | Western blot (human) | no | 29526398 |
| SEMG1 | Microarray and RT-PCR (human) | yes | 23289976 |
|  | Flow cytometry (human) | yes | 20720384 |
|  | Western blot and RT-PCR (human) | yes | 30575326 |
| TEKT1 | Western blot (human) | yes | 20304782 |
|  | Knock out mouse model | yes | 41764189 |
